# Supramolecular Carotenoid Complexes of Enhanced Solubility and Stability—The Way of Bioavailability Improvement

**DOI:** 10.3390/molecules24213947

**Published:** 2019-10-31

**Authors:** A. Ligia Focsan, Nikolay E. Polyakov, Lowell D. Kispert

**Affiliations:** 1 Department of Chemistry, Valdosta State University, Valdosta, GA 31698, USA; 2 Institute of Chemical Kinetics and Combustion, 630090 Novosibirsk, Russia; polyakov@kinetics.nsc.ru; 3 Institute of Solid State Chemistry and Mechanochemistry, 630128 Novosibirsk, Russia; 4 Department of Chemistry, The University of Alabama, Box 870336, Tuscaloosa, AL 35487, USA; lkispert@ua.edu

**Keywords:** carotenoids, inclusion complexes, delivery systems, nanoemulsions, nanoliposomes, biopolymeric nanoparticles, water solubility, oxidation stability, photostability, bioavailability

## Abstract

Carotenoids are natural dyes and antioxidants widely used in food processing and in therapeutic formulations. However, their practical application is restricted by their high sensitivity to external factors such as heat, light, oxygen, metal ions and processing conditions, as well as by extremely low water solubility. Various approaches have been developed to overcome these problems. In particular, it was demonstrated that application of supramolecular complexes of “host-guest” type with water-soluble nanoparticles allows minimizing the abovementioned disadvantages. From this point of view, nanoencapsulation of carotenoids is an effective strategy to improve their stability during storage and food processing. Also, nanoencapsulation enhances bioavailability of carotenoids via modulating their release kinetics from the delivery system, influencing the solubility and absorption. In the present paper, we present the state of the art of carotenoid nanoencapsulation and summarize the data obtained during last five years on preparation, analysis and reactivity of carotenoids encapsulated into various nanoparticles. The possible mechanisms of carotenoids bioavailability enhancement by multifunctional delivery systems are also discussed.

## 1. Introduction

**Figure 1:**
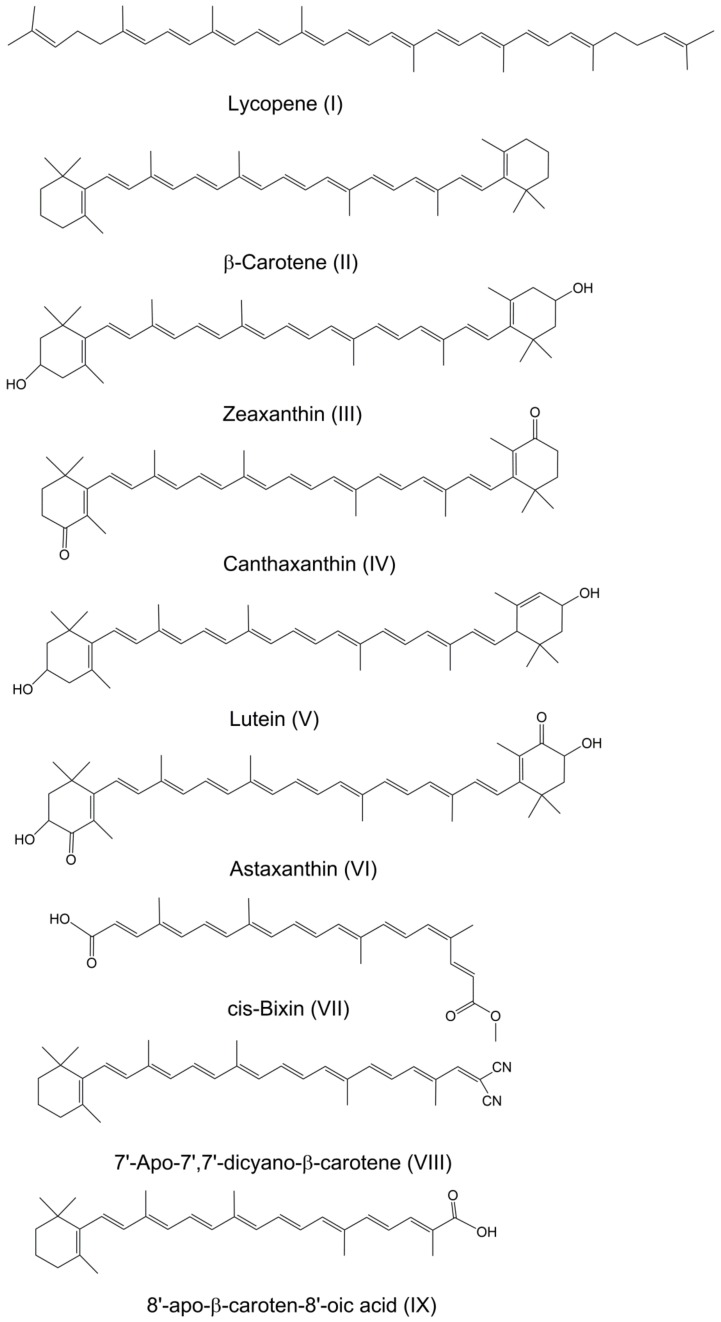
The ground state structures of selected natural and synthetic carotenoids **I**–**IX**.

Carotenoids are a class of bioactive compounds widely found in nature. These essential nutrients and pigments are synthesized by plants and microorganisms, and confer yellow, orange and red color to vegetables, fruits, and fish. The structures of most known natural compounds including β-carotene, canthaxanthin, lutein, zeaxanthin, lycopene, astaxanthin and bixin are illustrated in Figure 1, along with a couple of synthetic carotenoids containing cyano or carboxyl groups.

Carotenoids are considered as health promoters and have an important role in human nutrition and health. For example, β-carotene shows pro-vitamin A activity [1], astaxanthin is a powerful antioxidant and also demonstrates anticancer activity [2,3,4], lutein and zeaxanthin provide protection of eyes from harmful UV-irradiation and reactive oxygen species [5] and so on. Bixin was U.S. Food and Drug Administration (FDA) approved as a suitable colorant for use in food, drugs and cosmetics in the 1938 Federal Food, Drug and Cosmetic Act and in the 1960 Color Additive Amendments. Back then, bixin was shown to provide enhanced shelf life. Bixin is used in cosmetic compositions combined with a lipid soluble UV filter to protect the human epidermis against UV radiation.

A recent study [6] shows that bixin exhibits the highest measured carotenoid oxidation potential (0.94 V) to date. The suggested scavenging ability of *cis*-bixin towards reactive oxidative oxygen species (ROS) such as ^•^OH, ^•^OOH or O_2_^•−^ was estimated to be 17 times higher than that of carotenoid astaxanthin with an oxidation potential of 0.768 V [6]. The scavenging ability of carotenoids increases nearly exponential with increasing oxidation potential [7], thus explaining bixin’s increased shelf life. Another study [8] has shown that bixin does not form H-aggregates in methanol/water mixtures like zeaxanthin and astaxanthin do, but rather it forms J-aggregates instead, resulting in a red shift in the absorption spectrum. The lack of H-aggregation was traced to the fact that the ground state geometry for bixin is *cis* (see the structure in Figure 1) and not *trans*, resulting in an unfavorable geometry for H-aggregate formation. A large blue shift caused by H-aggregation would allow more photochemistry and could possibly lead to faster decomposition of the carotenoid. In a recent storage stability study [9], polar solvents like acetone and methanol gave a darker bixin color compared to water but the deterioration rate of the bixin in these solvents was faster as compared to water. Bixin in water retained its colour after 25 days at room temperature, at 4 °C and during sunlight exposure, indicating that water was the most stable solvent to prevent fast deterioration [9]. The enhanced water solubility of bixin was attributed to its *cis* ground state configuration [8]. However, most of the carotenoids in nature preferably occur as *trans* isomer hydrocarbons in their natural forms (Figure 1), and they possess extremely low water solubility. The hydrophobicity of carotenoids restricts wide practical applications with physiological benefits. In addition to their low water solubility, carotenoids possess chemical instability, being prone to oxidation. They contain polyene chains with up to 15 conjugated double bonds and this chemical feature is responsible for their instability to light, high temperature, oxygen and metal ions or interactions with radical species [10,11]. The sensitivity to the sun light and high temperature is very important from the point of view of storage stability of carotenoids and their application in cosmetics and the food industry. It appears that the low photostability of carotenoids is linked to the formation of carotenoid radicals. Carotenoid radical cations are formed by photoinduced electron transfer to electron acceptors [12] and their deprotonation in the presence of water molecules as proton acceptors creates carotenoid neutral radicals which are highly reactive species that can form a set of oxidation products as well as carotenoid dimers. Another proposed role of carotenoid neutral radicals is quenching of excited states of chlorophyll [12]. On the other hand, reactions with metal ions, mainly with Cu^2+^ and Fe^3+^, and ROS, accelerate the metabolism of carotenoids reducing their bioavailability. It is known that 1–3% of the adsorbed oxygen in the lungs is used for energy production and ROS generation which produces very reactive free radical species such as hydroxyl radicals [13].

Thus, developing methods for increasing bioavailability of carotenoids and stability towards irradiation and reactive oxygen species is an important goal. It was demonstrated that incorporation of carotenoids into “hosts” resulting in noncovalent binding between the nonpolar carotenoid and the water soluble “host” can significantly affect the physical and chemical properties of carotenoids [14]. For example, noncovalent binding of carotenoids with arabinogalactan (AG) or β-glycyrrhizic acid (GA) enhanced their water solubility and oxidation stability. Such inclusion complexes of carotenoids with arabinogalactan have shown enhanced photostability by a factor of 10 in water solutions, and a significant decrease by a factor of 20 in the reactivity towards metal ions (Fe^3+^) and reactive oxygen species in solution [15]. Complex formation decreased the rate of electron transfer from carotenoids to electron acceptors (Fe^3+^) [16]. The complexation with β-glycyrrhizic acid affected the oxidation potentials resulting in increased antioxidant activity of selected carotenoids [17]. Also, such inclusion complexes prevented aggregation of xanthophyll carotenoids in aqueous solutions [14]. H-aggregation of xanthophyll carotenoids detectable as 100 nm blue shift in the optical absorption maximum significantly changes the photophysics by increasing the quantum yield of reactive excited triplet state and reduces the scavenging ability of these carotenoids towards ROS. In addition, a remarkable increase in the quantum yield and the lifetime of the charge-separated states of the carotenoid radical cations in the supramolecular “hosts” β-glycyrrhizic acid and arabinogalactan has been detected [18].

Encapsulation of lipophilic carotenoids with different delivery systems, already done in numerous studies, is an innovative approach shown to increase their solubility, stability and bioavailability. Another advantage of encapsulation of bioactive compounds into nanosized delivery systems is their controlled release in human body [19,20]. Different delivery systems such as inclusion complexes, or nanoemulsions, nanoliposomes, and biopolymeric nanoparticles have been tested to improve carotenoid properties. This review covers basic concepts of nanoencapsulation of carotenoids and discusses recent developments that show the potential of nanoencapsulation technology.

## 2. Inclusion Complexes

Molecules of oligosaccharides and polysaccharides such as cyclodextrins (CD), glycyrrhizic acid (glycyrrhizin, GA) and arabinogalactan (AG) are capable of forming “host-guest” complexes with bioactive compounds substantially enhancing solubility and bioavailability [21]. Additional advantages like improvements in chemical stability, protection of bioactive compounds from external environment, taste modification and controlled release make them interesting delivery systems for further study to incorporate bioactive compounds into beverages or foods fortified with bioactive compounds, or in pharmaceuticals or cosmetics.

### 2.1. Cyclodextrins

**Figure 2:**
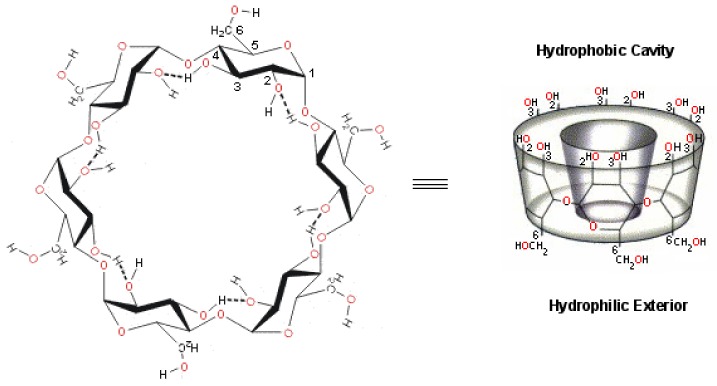
The cyclic oligosaccharide cyclodextrin (CD).

Cyclodextrins (CD, Figure 2), cyclic oligosaccharides derived from starch, are the most popular and developed drug delivery systems [22,23]. CDs have a hydrophilic shell and hydrophobic core suitable for the inclusion of lipophilic “guest” molecules of appropriate size. They are widely applied in the pharmacy and the food industry to enhance the aqueous solubility and dissolution rate of low soluble bioactive molecules, as well as for increasing their stability and bioavailability [24]. Using CDs to encapsulate carotenoids can also reduce light and heat-induced degradation and oxidation. CDs are inexpensive, non-toxic, they possess low hygroscopicity and high thermal stability [25].

The most common CDs are α-CD, β-CD and γ-CD, which have different numbers of glucopyranose units—6, 7 and 8 units—and different ring diameters of about of 6, 8 and 10 Å, respectively. Different CDs have different solubility in water, and their stability and controlled release can be changed by altering their structure, for example by changing the ring size or adding groups [25]. For instance, the water solubility increases significantly by chemical modification of the most low soluble β-CD to produce hydroxypropyl- or methyl-substituted β-CD [24,25]. Enhanced storage stability was also shown in earlier studies that involved the carotenoid/CD inclusion complexes [26]. The complex of astaxanthin with hydroxypropyl-β-CD showed storage stability and antioxidant activity [26] but poor solubility. 

The encapsulated hydrophobic carotenoids substitute the enthalpy rich water molecules within the cavity without cleavage or formation of covalent bonds, and they remain in the hydrophobic interior via hydrophobic forces, van der Waals interactions or hydrogen bonds [25]. Non-covalent associations of *trans*-β-carotene complexes with β- CD and γ-CD in water showed evidence for the formation of large aggregates by light scattering and NMR spectroscopy [27]. A study on the lycopene/α- and β-CD complexes by light scattering, ion spray ionization and tandem mass spectrometry also pointed out that large aggregates of particles, on the nanometer-size scale, were present in water, with meaningful differences in the shape when comparing the α-CD with β CD aggregates [28]. ^1^H-NMR, EPR and optical studies on inclusion complexes of carotenoids with cyclodextrins showed that CD protects the carotenoid from reactive oxygen species but complexation with CD results in considerable decrease in antioxidant ability of the carotenoid [29]. In reality, these complexes form water dispersions, rather than solutions. The reduced color intensity significantly decreases the use of carotenoid/CD complexes, in particular as food colorants. 

Nevertheless, some positive results have been obtained during the last years. In particular, a recent study of carotenoids/CD complexes shows that molecular inclusion of yellow bell pepper carotenoids (lutein, zeaxanthin, α-cryptoxanthin, α-carotene and β-carotene) into the CD cavity provides good results for color protection for beverages compared with the use of the crude extract [30]. The authors have measured the color stability during storage under irradiance and in the absence of light at temperatures between 25 and 31 °C for 21 days and demonstrated higher stability of the color indexes in isotonic drinks colored with the inclusion complexes compared to colored with crude yellow bell pepper extract. Several carotenoids/β-CDs inclusion complexes provided evidence that β-CDs inclusion renders carotenoids more stability towards oxidizing agents like 2,2’-azobis(2-methylpropionamidine) dihydrochloride (AAPH) and hydrogen peroxide (H_2_O_2_) [31]. Pinzón-García and coauthors [32] investigated the effects of oral administration of the antioxidant carotenoid bixin and its β-CD complex in an obese murine model. The authors conclude that the oral administration of the bixin/β-CD inclusion compound improved the metabolic parameters, being more palatable and hepatoprotective. In another study, Nalawade and Gajjar [33] showed an improved dissolution rate of carotenoid astaxanthin by its complexation with methyl-β-CD using spray drying technique. Application of various physicochemical techniques, FTIR, UV, DSC, ^1^H-NMR, XRD and molecular modeling analysis, confirmed that the side rings of astaxanthin molecule were incorporated into the CD cavity. This study provides the basis for the development of soluble and bioavailable oral formulations of carotenoid complexes with cyclodextrins using spray drying technique, which is scalable and accepted in the industry.

### 2.2. Glycyrrhizic Acid

**Figure 3:**
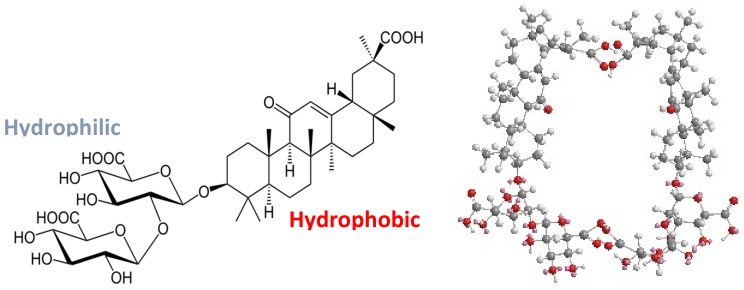
The structures of glycyrrhizic acid monomer and dimer.

Glycyrrhizic acid (GA) is a saponin extracted from licorice root. Like most saponins, the GA molecule has an amphiphilic structure. It contains a hydrophilic part— a glucuronic acid residue—and a hydrophobic part—a glycyrrhetic acid residue (see Figure 3). 

Application of GA in medicine has very long history [34,35]. Licorice has been used in traditional medicine since ancient times in China, Egypt and Japan [34]. Nowadays, the biological and therapeutic activity of GA is still intensively investigated, and it was confirmed to be non-toxic for application in medicine and food industry [34,36,37].

Only 15 years ago a novel unusual property of the GA has been discovered, namely its ability to form stable self-associates [38,39,40] which are able to form water-soluble inclusion complexes with other lipophilic molecules. In particular, it was found that glycyrrhizin is able to form water-soluble supramolecular complexes with a variety of lipophilic drugs [35,41,42,43,44,45,46,47,48,49,50,51,52,53] These complexes show significant advantages over other known drug delivery systems, possessing increased solubility, stability and bioavailability. In addition, the membrane modifying ability of GA has been described [21,54,55,56,57]. We consider that the key factor in its therapeutic activity is its ability to incorporate into the lipid bilayer and to increase the membrane fluidity and permeability [35].

Supramolecular complexes of β-carotene, canthaxanthin, lutein, zeaxanthin, astaxanthin and other carotenoids with GA have been prepared, and their properties were studied by various physicochemical methods (NMR, EPR, electrochemistry, UV spectroscopy) [14,16,17,58,59,60,61]. In these studies, carotenoid-GA complexes have been prepared by two different methods. First method was traditional “liquid state” technique. Since GA acid is soluble both in water and organic solvents, a solution of carotenoid in methylene chloride was added to the aqueous or organic GA solution and then stirred for several hours for complex preparation. The second “solid state” method of complex preparation was a mechanochemical treatment of the solid mixture of carotenoid crystals with GA crystals. Co-grinding of powder components results in formation of solid dispersion without use of any organic solvents. In such processes hydrogen bonds, π-stacking, van der Waals, ion pairing interactions etc. are broken leading to formation of supramolecular complexes directly in solid state or hybrid molecular crystals.

Several important properties of carotenoids-GA complexes were found in these studies:

(1) Carotenoids are able to form inclusion complexes with GA micelles at high concentrations (>1 mM of GA), as well as with pre-micellar GA aggregates (dimers) at low concentrations (1 µM–1 mM) [16]. These complexes are extremely stable. The stability constants of carotenoids-GA complexes are 1–2 orders higher than stability constants of carotenoids-CD complexes.
(2) Encapsulation of carotenoids lutein and zeaxanthin into GA micelles protects these carotenoids from oxidation by reactive oxygen species (O_3_ and OH radical) and metal ions [60]. For example, oxidation rate of lutein and zeaxanthin by ozone molecules in aqueous-ethanol solution decreased 10 times in the presence of 1 mM of GA. Similar effects were detected in the presence of disodium salt of GA: in the presence of 1 mM of Na_2_GA the oxidation rate of lutein and zeaxanthin by Fe^3+^ ions decreases by 10–20 times [60].
(3) In contrast to most of water soluble oligosaccharides and polysaccharides [14,58], GA is able to form supramolecular complexes with carotenoids not only in aqueous solutions where GA complexes increased the carotenoid solubility more than 1000-fold [60], but also in non-aqueous organic solvents (alcohols, DMSO, acetonitrile) [14,16,58]. This fact is important for discussion the possibility of GA-assisted transport of carotenoid molecules through lipophilic cell membranes and their membrane protection properties.
(4) In organic solvents GA is able to form supramolecular complexes not only with neutral carotenoid molecules, but also with their paramagnetic forms-radical cations and charge transfer complexes with electron donors [16]. Also, there is a significant increase of the lifetime of β-carotene radical cations (50-fold) in the presence of GA [16]. High stability of the carotenoid radical cations imbedded into GA host opens possibilities for the application of these complexes for the design of artificial light-harvesting, photoredox and catalytic systems.
(5) One of the most important biological properties of carotenoids is their antioxidant activity. In aqueous environment as well as in lipid membranes carotenoids trap toxic oxygen radicals and thus prevent damage to living organism [62,63]. Some studies were performed to elucidate how the complexation with GA affects the ability of carotenoids to scavenge reactive oxygen radicals [17,59]. The antioxidant activity of carotenoid complexes was studied by the EPR spin-trapping technique. The details of this technique and effectiveness in the measurement of scavenging rates towards hydroperoxyl OOH radical are described in our earlier study [7]. Comparison of the scavenging rates of hydroperoxyl radicals by free carotenoids and their GA complexes in non-aqueous solution (DMSO) shows a strong dependence of the rate constants on the carotenoids structure and their oxidation potentials (see Table 1).

In a recent study the oxidation potential of *cis*-bixin referenced to ferrocene (0.94 V) was listed as the highest measured oxidation potential of all carotenoids studied [6]. Using this oxidation potential and extrapolating the scavenging ability plot predicts a relative scavenging value for *cis*-bixin of 44 or a value of 501 assuming a semi log plot [6]. This sets bixin as the naturally occurring carotenoid with the highest scavenging rate, even higher than that of the synthesized 7-*apo*-7,7-dicyano-β-carotene. The carotenoid 7-*apo*-7,7-dicyano-β-carotene was found to be very stable when tuning lasers in Fleming’s lab at U. of California, Berkeley. Similarly, we expect bixin to be very stable. Along with its highest scavenging ability and its potential to increase shelf life, bixin makes a great candidate for being used as a dye in foods, drugs or cosmetics. Furthermore, very little amounts are needed when used as coloring agent because *cis*-bixin has a high extinction coefficient of 10^5^ [64].

Since it was found [17] that scavenging ability of carotenoids towards hydroperoxyl radicals is strongly potential dependent, it was suggested that GA complexation can affect the oxidation potential of the carotenoids. This hypothesis was proved by the measurement of the oxidation potential of two carotenoids zeaxanthin and canthaxanthin in the presence of GA. In both cases, an increase in E_1/2_ by 0.03-0.05 V was observed [17]. It was assumed that interaction between carotenoids and hydroperoxyl radicals occurs via hydrogen abstraction from the most acidic 4-H proton of carotenoids [59,65]. GA forms a donut-like dimer in which the hydrophobic polyene chain of carotenoids lies protected within the donut hole, permitting the hydrophilic ends and most acidic proton to be exposed to the surroundings. Note that in the case of the cyclodextrin complexes, the terminal group of the carotenoid is completely protected which results in the inhibition of antioxidant activity [29].

The characteristic feature of xanthophyll carotenoids (lutein, zeaxanthin, astaxanthin, β-cryptoxanthin) is their ability to self-assemble into J- and H-type aggregates in aqueous solution and in lipid membrane [59,66]. The formation of such aggregates occurs even in organic solvents in the presence of small amount of water (5–10%) and significantly changes the photophysical and optical properties of these carotenoids which are important for solar energy conversion and light induced oxidative damage [59,67]. As an example, in the absorption spectrum of zeaxanthin, a large shift of absorption band from 460 nm with appearance of the vibrational bands to 380 nm with the loss of vibrational structure of the S2 excited state indicates the presence of the zeaxanthin dimers. In contrast, the optical study of *cis*-bixin [8] shows minimal problem with aggregation in mixtures contrary to some *trans* carotenoids like zeaxanthin. Due to the unfavorable *cis* ground state structure there is lack of H-aggregation but there is a 4 nm red shift in a 50% water-methanol solution, suggesting J-aggregation. The optical shifts with solvent depend on the polarity of the solvent. Lack of H-aggregation prevents a large blue shift, which would allow for more blue light absorbance, possibly causing decomposition. Also, the antioxidant activity of xanthophyll carotenoids significantly reduced due to aggregation reaction [59]. This feature might be important for antioxidant and photoprotective function of these carotenoids in lipid membranes [68]. In particular, it was shown by the EPR technique that in the presence of Fe^2+^ ions and hydrogen peroxide (Fenton reaction) zeaxanthin aggregates can demonstrate pro-oxidant effects instead of antioxidant activity [59]. It was demonstrated by UV spectroscopy that complexation with GA reduces the aggregation ability of the xanthophyll carotenoids [59]. Simultaneously, the scavenging ability of xanthophyll carotenoids to trap free oxygen radicals increases since GA prevents aggregation allowing the cyclohexene ring of carotenoids to be exposed to the surroundings. Considering the important role of xanthophylls in eye and skin health, glycyrrhizin should be considered as a perspective delivery system to provide enhanced solubility and activity of the carotenoids.

Recent studies have shown that GA is able not only to form inclusion complexes with carotenoids and various drug molecules, but also to modify the structure of lipid membranes [21,34,54,55,56,57]. Using NMR relaxation techniques and molecular dynamics simulation (MD) it was demonstrated that GA molecules are able to penetrate into lipid bilayer and affect the lipid dynamics. Taking into account the protective role of some carotenoids in lipid membranes [68,69,70], and the ability of GA to form stable associates with carotenoids in non-aqueous environment, one can expect the influence of GA on the antioxidant ability of carotenoids in membranes. In this case the ability of GA to prevent the xanthophyll carotenoids aggregation and thus to enhance their antioxidant properties [59] also can play certain positive role.

### 2.3. Arabinogalactan 

**Figure 4:**
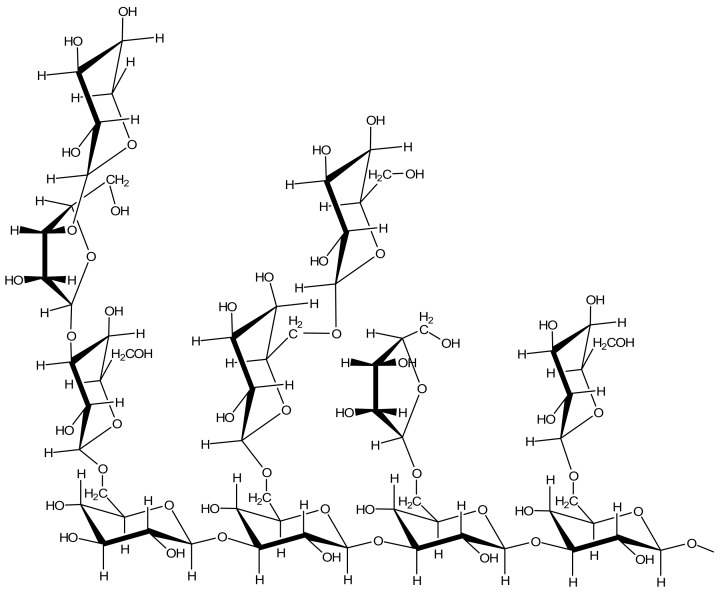
Fragment of a branched polysaccharide arabinogalactan (AG).

The next “host molecule” for carotenoid encapsulation and delivery described in the literature is polysaccharide arabinogalactan (AG, Figure 4). AG is a natural branched polymer consisting of arabinose and galactose units with a molecular mass near 16 kDa extracted primarily from Western and Siberian larch [71,72].

It is highly water-soluble and produces low-viscosity solutions. Larch arabinogalactan is non-toxic and approved by the FDA as an important source of dietary fiber. Arabinogalactan is biodegradable, biocompatible and hydrophilic, contains different functional groups such as hydroxyl, carboxylic acid, that make it ideal for conjugation and delivery of carotenoids and drug molecules. There are many examples of increased solubility of hydrophobic drugs in the complexes with AG [72,73,74]. Some recent studies indicate also that AG has good adhesion to membrane surface, and is able to enhance the membrane permeability for both human and plant cell membranes [21,75].

Since AG is soluble only in water solution, but carotenoids are insoluble in water, the solid state mechanochemical technique had been developed for preparation of carotenoid-AG inclusion complexes directly in solid state without using of any organic solvents [15,59,60,61,76]. Co-grinding of the solid polysaccharide with carotenoids results in penetration of the carotenoid molecules into the “host” polysaccharide, and formation of a water-soluble carotenoid complex. As a result, the solubility of β-carotene and cantaxanthin complexes with AG prepared for the first time [15] were 2–5 mM in aqueous solution, which is six orders of magnitude higher than the characteristic solubility of free carotenoids in pure water (~1 nM [77]). The mechanochemical method for solid-state complex preparation has evident advantages compared with traditional “liquid phase” techniques. The interest in solvent-free conditions stems from the possibility of producing the same product as that from solution without solvent because this process is cheaper, less time consuming and more environmentally friendly. In the case of carotenoids, the solid state technique opens the possibility of obtaining products not otherwise accessible in solutions.

As it was mentioned in the Introduction, one of the main problems with the practical application of carotenoids is their chemical instability in the presence of light, oxygen, water and metal ions. Although they are considered as effective photoprotectors of living cells, in aerated aqueous solution they are unstable and would be unacceptable as colorants and antioxidants in foods. Various physicochemical techniques have been applied to study the reactivity of carotenoid incorporated into AG macromolecule in aqueous solution. The carotenoid-AG complexes maintain their original color and show insignificant changes in absorption spectra [15,59]. It was demonstrated also that complexation with AG prevents H-aggregates formation of xanthophyll carotenoids in the presence of water as it was detected for carotenoid complexes with GA [59]. It was found that AG complexes have enhanced photostability and oxidation stability compared to pure carotenoids. We suggest that these results are important for a variety of carotenoid applications. A significant increase (5-10 times) in photostability of carotenoids lutein, astaxanthin and canthaxanthin in the inclusion complex with AG was detected [15,59].

**Figure 5:**
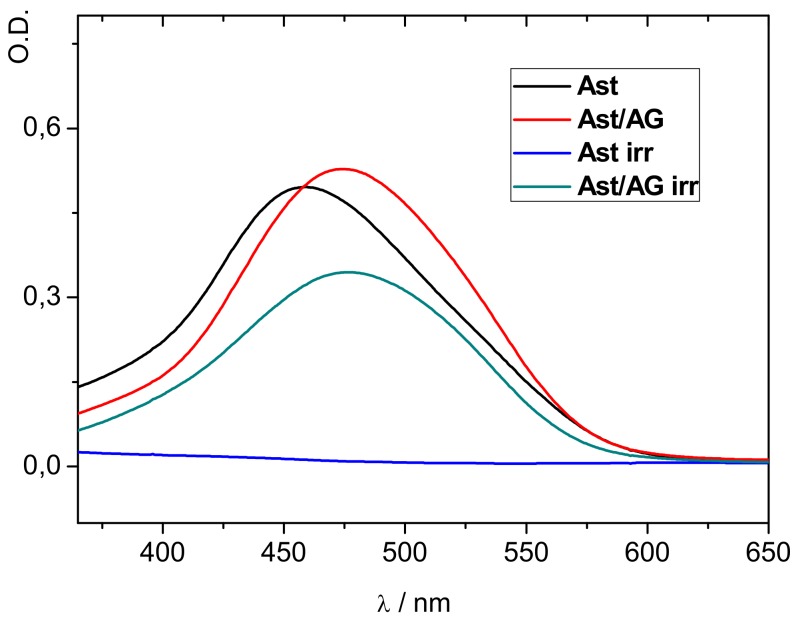
Photodegradation of astaxanthin in aerated 25% ethanol solution by the full light of a xenon lamp. Irradiation time 1 min (adopted from [59]).

As an example, Figure 5 demonstrates the difference in the photodegradation rate of astaxanthin in pure form and in the complex with arabinogalactan ([AG] = 0.1 mM) in the presence and in the absence of irradiation. The authors proposed that the main mechanism of enhanced photo stability of carotenoids in a polysaccharide complex is their isolation from water by incorporation into the hydrophobic polymer environment. 

**Figure 6:**
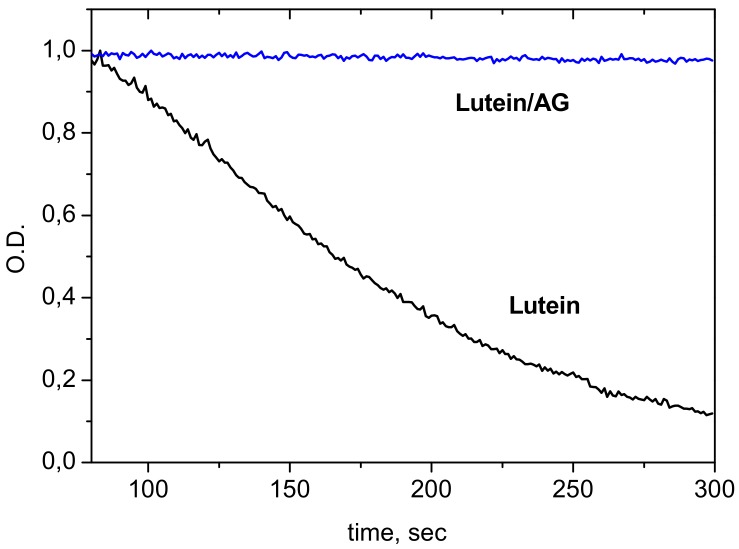
Oxidation of lutein and lutein/AG complex by ozone in 25% ethanol solution. Concentration of AG 0.05 mM (adopted from [60]).

Incorporation of carotenoids into the hydrophobic polymer environment results also in enhancement of their chemical stability. The complete inhibition of oxidation of the carotenoids lutein and zeaxanthin by Fe^3+^ ions as an electron acceptor and by ozone molecules as powerful oxidant was achieved by complexation with AG [60]. Oxidation of carotenoids by Fe^3+^ ions results in formation of carotenoid radical cation, and this reaction was described in details in our earlier studies [16,78]. On the other hand, radical cations of carotenoids are unstable in aqueous solutions due to fast deprotonation and formation of carotenoid neutral radicals [79]. Figure 6 demonstrates an example of the difference in the oxidation rate of lutein by ozone in pure form and in the complex with AG in aqueous solution. 

**Scheme 1:**
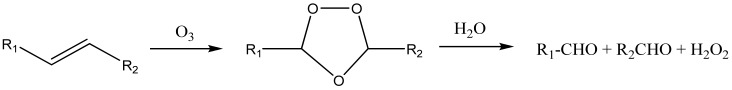
The mechanism of decay the conjugated double bonds of carotenoids and other unsaturated compounds in the presence of ozone in water solution [81].

Ozone molecules are known to react with unsaturated double bonds with high efficiency [80,81]. The products of this reaction are unstable ozonides which decay to the final oxidation products—ketones, aldehydes, and carboxylic acids. As an example, Scheme 1 shows the reaction pathway in aqueous solution.

The authors [60] suggested two main factors responsible for chemical stabilization of carotenoids in AG complexes, namely, isolation from water and isolation from reactive species (ozone molecules and metal ions). We assume that the stability of the carotenoids incorporated into the polysaccharide macromolecule might have wide practical application.

**Figure 7:**
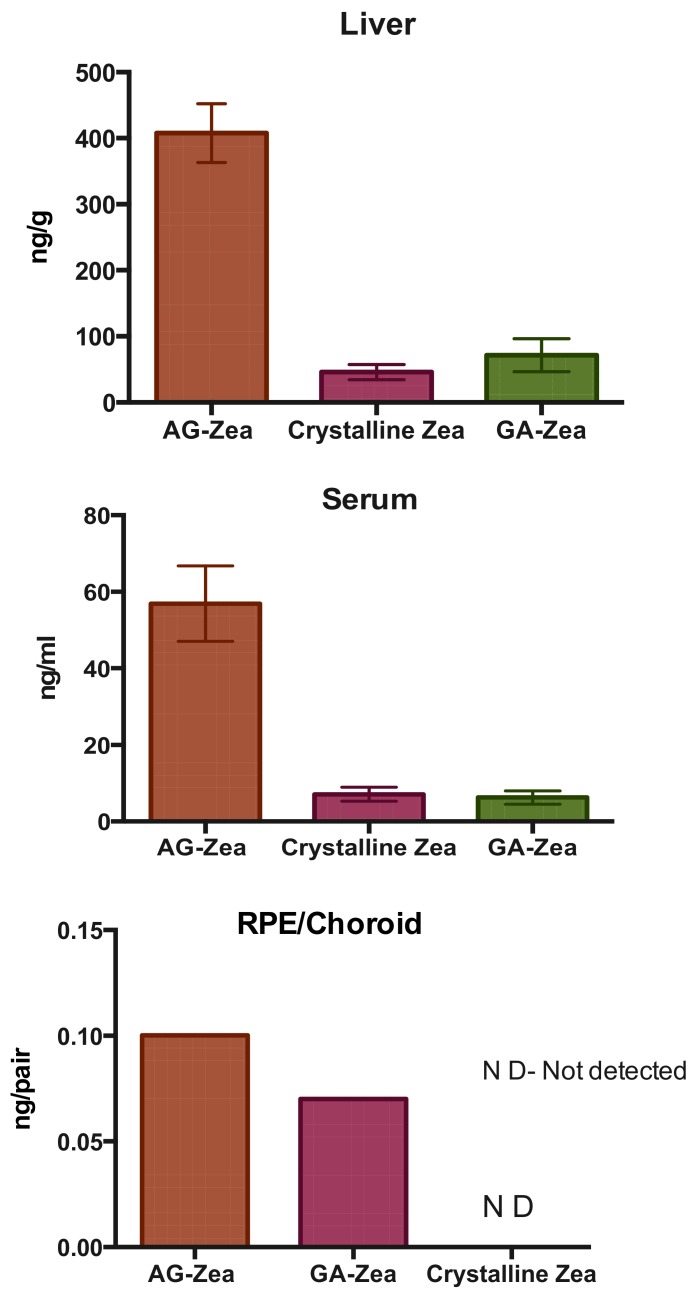
Comparison of three carotenoid delivery methods in C57BL/6 wild type (WT) mice. 12-week-old WT mice were fed with chow containing zeaxanthin in the formulations of crystalline carotenoid, complex with glycyrrhizic acid (1:20) and complex with arabinogalactan (1:20). Values indicate means ± SD from 5 to 10 mice in each group. Retinal pigment epithelium (RPE) is the pigmented cell layer just outside the neurosensory retina that nourishes retinal visual cells, and is firmly attached to the underlying choroid and overlying retinal visual cells (adopted from [61]).

An important question is whether the described features of the complexes (solubility, membrane permeability, stability) affect the bioavailability of carotenoids in living systems. To answer this question, Li and coauthors [61] investigated the retinal accumulation of macular carotenoids in wild type mice when fed with zeaxanthin and lutein using an optimized carotenoid feeding method. Macular carotenoids are well-known natural antioxidants and light screening compounds that are capable of quenching singlet oxygen and other free radicals and absorbing potentially damaging blue light [82]. Supplementation with macular carotenoid can prevent and reduce the risk of age-related macular degeneration and other ocular disease. The authors performed HPLC analysis revealed that zeaxanthin and lutein were detected in serum, liver and RPE/choroid of the mice. Significantly higher amounts of lutein and zeaxanthin were detected when they use carotenoid/AG complex instead of crystalline carotenoids. Interestingly, only the GA and AG complexes are able to successfully deliver zeaxanthin into the RPE/choroid of WT mice (see Figure 7). 

We can conclude that AG and GA are perspective delivery systems for various carotenoid applications. These complexes demonstrated enhanced solubility and stability in aqueous solutions, as well as enhanced bioavailability in model animal studies. Table 2 summarizes the delivery systems, carotenoids, encapsulation methods and results with references for studies using CD, GA and AG as delivery systems.

## 3. Biovailability of Carotenoids and Lipid-Based Nanocarriers

The bioavailability of carotenoids in foods, nutraceuticals and pharmaceuticals varies greatly, depending on a multitude of factors such as the food matrix or the type of delivery system, the type of carotenoid (polar versus nonpolar), the fate of the carotenoid during processing and in digestion, etc. Generally, the carotenoid is first emulsified, then solubilized in micellar form within the intestine with an absolute requirement for conjugated bile salts. For emulsification, the carotenoids have to be incorporated into oil droplets, either formed during lipid digestion or present in the original food (for example, emulsions). During the process of digestion, carotenoids are incorporated with other lipids into mixed micelles (also containing bile salts and phospholipids), which behave as carriers to solubilize the carotenoid and transport it to the intestinal absorptive cells. Carotenoids are incorporated by intestinal mucosal cells into chylomicra, a lipoprotein formed in the small intestine that transports dietary fats and cholesterol through the lymphatic system to the bloodstream. In plasma, carotenoids are transported by very low density lipoproteins (VLDL) and low density lipoproteins (LDL) as well as high density lipoproteins (HDL).

**Figure 8:**
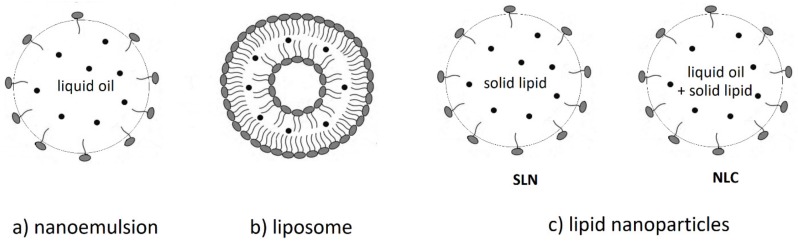
Lipid-based nanocarriers: (**a**) O/W nanoemulsion: Liquid oil in continuous aqueous phase stabilized by surfactant (amphiphilic molecule, in gray). Carotenoid molecules are shown as black dots. (**b**) Liposome: concentric phospholipid bilayer with a hydrophilic center. Carotenoids shown as black dots are imbedded in the hydrophobic area of the bilayer. (**c**) Lipid nanoparticles: Solid lipid nanoparticles (SLNs) are constituted only by solid lipids, whereas nanostructured lipid carriers (NLCs) are a mixture of mainly solid with liquid lipids. Carotenoid molecules are shown as black dots.

The bioavailability of carotenoid is partially determined by its bioaccessibility, which is generally defined as the fraction of the ingested carotenoid that is incorporated into the mixed micelles and thus becomes available for absorption in the body [83]. Mixed micelle formation upon lipolysis of the emulsified triglycerides in the presence of bile acids and phospholipids is essential to the transfer of the water insoluble carotenoid to the intestinal epithelia for uptake into the body. Simultaneous consumption of carotenoids with digestible lipids promotes their bioaccessibility and bioavailability. The presence of lipids simplifies the absorption of carotenoids since it increases the possibility of formation of the mixed micelles for solubilization and transport. In this regard, application of lipid based delivery systems has been explored in different studies. In the past few years, lipid-based nanocarriers have been recognized as one of the most efficient approaches to encapsulate carotenoids [84,85]. The main types of lipid-based nanocarriers for carotenoids (see Figure 8) that have been studied and developed in recent years (see examples of studies in Table 3, Table 4, Table 5 and Table 6) include nanoemulsions (typically O/W), nanoliposomes, SLNs and NLCs.

### 3.1. Nanoemulsions

Nanoemulsions are appropriate delivery systems that have shown promising results for encapsulation of poorly soluble carotenoids (see Table 3 for a summary of recent studies on nanoemulsions). Emulsions are dispersions that consist of oil, surfactants, and aqueous phase. Due to the similarities between microemulsions and nanoemulsions, various definitions have been proposed in the literature [86]. One of them defines nanoemulsions as “transparent or translucent systems containing droplets with a mean diameter in the range between 100–500 nm, and unlike thermodynamically stable microemulsions, they are kinetically stable”. Nanoemulsions appear optically transparent due to their very small droplet sizes, and have diameters usually between 50 nm and 250 nm [25,87] although different size ranges have been reported in literature, some up to 600 nm [88]. The core of the nanoemulsion particle can be either oil or water (the O/W or W/O nanoemulsion, respectively). The oil-in water (O/W) nanoemulsion (see Figure 8a) which consists of disperse-phased oil droplets in a continuous aqueous phase stabilized by surfactants (or emulsifiers) has received interest due to its high stability, aqueous solubility, high bioavailability and ease of processing.

Different bioactive oils can be used in combination with hydrophobic carotenoids as the lipid phase. The viscosity and surface tension of the oil phase influences the droplet size. Smaller droplet sizes are produced by lower viscosity and interfacial tension of the oil phase, while larger droplet sizes are produced by high viscosity and interfacial tension oil phase like in long chain triglycerides [88]. Oils containing long chain triglycerides are mostly used, but medium-chain or even short-chain can be used to make nanoemulsions. Edible oils are preferred because during digestion, the triacylglycerol oils break down within the gastrointestinal tract to form the mixed micelles that are capable of solubilizing and transporting nonpolar bioactive components [84]. Formulating nanoemulsions with specific structures and compositions to boost bioavailability and maintain bioactivity requires understanding the fate of health-related food components in the gastrointestinal tract [89].

Corn oil, a source of long chain triacylglycerols, has been shown to increase the bioaccessibility of carotenoids. However, the content of oil was demonstrated [90] to be important in the preparation of O/W nanoemulsions to encapsulate β-carotene in a form where the fat phase is fully digested. The low-fat (4%) content was fully digested, whereas the high-fat (20%) content was only partially digested. The micellarization of β-carotene was higher in the high-fat group, however the bioaccessibility of the high-fat group (39%) was much lower than that of the low-fat group (84%), which was attributed to a fraction of the carotenoids remaining in the nondigested lipid phase of the high-fat group. Also, it is believed that another factor that influences lipid digestion and carotenoid bioaccessibility is the droplet size of nanoemulsion. A smaller droplet size with higher surface area would facilitate higher transfer of lipophilic bioactive compounds into micelles. The rate and extent of lipid digestion and β-carotene bioaccessibility increased with decreasing mean droplet diameter. As the droplets passed through a simulated gastrointestinal tract (GIT) the initial size droplet increased, fact attributed to droplet coalescence, flocculation, and digestion. Thus, the smallest initial droplet size produced the best result for the bioaccessibility of the carotenoid [91].

Another study [92] showed that although dietary fat significantly increases the micellarization of carotenoids from all test foods, the extent of carotenoid micellarization depends on the type of dietary fat and the polarity of carotenoids, along with the food matrix. A general trend of higher micellarization was observed for dietary fat rich in unsaturated fatty acids (olive oil = soybean oil = sunflower oil) versus saturated fatty acids (peanut oil = palm oil > coconut oil), and lutein being absorbed better than other nonpolar carotenoids (lutein > β-carotene = α-carotene > lycopene). A recent study [93] concluded that palm oil (compared to corn oil, fish oil and coconut oil) was the most suitable to be used as the lipid phase in formulation of O/W nanoemulsion to increase bioaccessibility and stability of β-carotene. As briefly described above, bioavailability of carotenoids, or the proportion that is digested, absorbed, and metabolized through normal pathway, depends on factors such as the type of the carotenoid, amount of carotenoid, the food matrix, fat type and fat content of the diet. There are numerous other factors but not limited to conversion of provitamin A carotenoids to vitamin A, absorption rate, transport, nutrient status of the host, genetic factors, host-related factors, interactions of these factors, and the complexity of its release in GI tract during digestion [94].

For encapsulating hydrophobic compounds in O/W emulsions, an emulsifier (also called surfactant, or stabilizer) is usually required. Nanoemulsions are thermodynamically unstable [95] and can be kinetically stabilized by adding an emulsifier. The emulsifier lowers the surface tension between water and oil phases, stabilizes the lipophilic components in the aqueous phase and increases the solubility. Emulsifiers have an amphiphilic structure with a hydrophilic or water-loving head and a hydrophobic or fat loving tail. The hydrophilic head protruding into the water phase and the hydrophobic tail into the oil phase of the emulsion, thus holding together ingredients that would not normally mix very well. An emulsifier can be a small molecule like polyoxyethylene sorbitan fatty acid esters also known as Tweens, a phospholipid, or a larger molecule such as a protein or a polysaccharide [96]. Tweens, such as Tween 20, 40, 60 and 80, which are commonly used to make emulsified delivery systems for hydrophobic nutraceuticals, are considered synthetic. There is an increasing demand for natural emulsifiers such as proteins, phospholipids, polysaccharides and saponins.

The most commonly used protein-based emulsifiers in the food industry today are caseins and whey proteins, which are both food-grade proteins present in milk [97]. Sodium caseinate (SC) is its most common form of casein and is a good alternative for synthetic surfactants because it shows good thermal stability and prevents oxidation of the carotenoid and the emulsified oils. Liu et al have used caseinate-stabilized emulsion as delivery system for astaxanthin. Solution pH, ionic strength and light exposure had little impact on the chemical stability of the carotenoid, except at pH 4 and 5. The emulsions showed thermal stability (5–70 °C) but the chemical degradation of astaxanthin increased with increasing temperature. At pH values near the isoelectric point of the caseinate (pH 4.6) the emulsions were highly unstable to droplet aggregation [98]. SC was also used as emulsifier in O/W emulsions containing β-carotene homogenized at different higher and lower pressures to give different droplet diameters (124–368 nm). The encapsulated β-carotene was more unstable towards oxidation as droplet diameter decreased with increasing pressure [99]. Whey protein used as emulsifier to form nanoemulsion for encapsulation of lutein showed promising result, with lutein content being reduced by only 4% after four weeks storage at 4 °C [100].

Phospholipid emulsifiers used in preparation of emulsions can be soybean lecithin, egg lecithin or lysolecithin [95]. The effect of modified lecithin (ML) and sodium caseinate (SC) on the formulation, stability and bioaccessibility of astaxanthin loaded O/W nanoemulsions was compared, and it was found that the bioaccessibility of astaxanthin in nanoemulsions was significantly higher in ML (33%) than in SC-stabilized nanoemulsions (6%). ML-stabilized nanoemulsions were stable for 30 minutes against a wide range of pH and heating temperatures (60–120 °C) but showed droplet growth when treated at high salt concentrations. On the contrary, SC-stabilized nanoemulsions were stable at high salt concentration and showed good physical and chemical stability after 30 days of storage [101]. 

Saponins, which are derived from plant materials and, in addition, possess pharmaceutical properties [102] are very well suited to be used as natural emulsifiers. Structurally, they contain a carbohydrate moiety attached to a triterpenoid or steroids [103]. Ginseng saponins (GS) are active ingredients in ginseng (the root of *Panax* medicinal plants), most of which are glycosides of triterpenoid aglycones [104]. Soybean O/W nanoemulsions stabilized with GS and loaded with astaxanthin were stable against thermal treatment (30–90 °C, 30 min), and over a narrow range of pH values (7–9), but unstable in acidic conditions (pH 3–6) and in the presence of salt [105]. Gypenosides (GP) are triterpenoid saponins that are traditionally used in herbal medicine. Gypenosides were investigated and compared with Tween 20 in formulating astaxanthin loaded O/W nanoemulsions [106]. GPs produced smaller droplets (125 nm) that were stable over a narrow range of pH values (6–8) and thermal treatments (60–120 °C) but unstable in the presence of acidic or salt conditions. GPs were more effective at inhibiting astaxanthin from degradation during 30 days of storage at both 5 and 25 °C but led to lower lipid digestion and bioaccessibility [106].

Combinations of two or more different emulsifiers may improve the formation, stability, and performance of O/W emulsions [107]. Traditionally, O/W emulsions use a single emulsifier or a combination of two or more emulsifiers, in which the oil droplets are surrounded by a single interfacial layer. A bilayer emulsion can be prepared by a layer-by-layer electrostatic deposition technique when an emulsifier or a polyelectrolyte is added to an already-formed monolayer emulsion and adsorbed on the droplet surfaces under certain pH. The thicker membrane coat outside the droplets can improve the stability of the bilayer emulsion. In their review, Sheng et al [108] have listed some examples of bilayer emulsions such as lecithin and chitosan, β-lactoglobulin and pectin, sodium caseinate and carboxymethyl cellulose, sodium caseinate and xanthan gum, whey protein isolate and oxidized starch adhesive, etc. The authors have developed a new type of bilayer emulsion delivery system with good physicochemical properties, comprising bovine serum albumin (BSA) as the inner emulsifier and Arabic gum as the outer emulsifier to encapsulate β-carotene [108]. The bilayer emulsion formed had improved properties such as small size and size distribution, strong potential, high viscosity, good physical stability, high encapsulation ratio of β-carotene, low interfacial tension and better chemical stability that could weaken the influence of low pH, thermal treatment, UV radiation, or oxidants.

### 3.2. Nanoliposomes

Liposomes are spherical vesicles made of concentric phospholipid bilayer with a hydrophilic center (see Figure 8). Liposomes can thus encapsulate hydrophilic substances into the interior part, hydrophobic substances within hydrophobic bilayer, or amphiphilic substances at the lipid/water interface [88]. Liposomes entrap carotenoids into the hydrophobic phospholipid bilayer (see Figure 8 and Table 4 for recent studies described in [109,110,111,112,113]). Nanoliposomes, the nano version of liposomes with mean diameter range approx. 50–150 nm [84], can improve the solubility and bioavailability of bioactive compounds, as well as their in vitro and in vivo stability, and they can target specific cells.

The most common ingredients to prepare nanoliposomes is phosphatidylcholine, a phospholipid that is often termed as lecithin because phosphatidylcholine is a major component of lecithin extract. Phosphatidylcholine can be easily obtained from a variety of readily available sources such as egg yolk or soybeans. Cholesterol is another lipid used to prepare liposomes, however, it increases the membrane rigidity and decreases the rate of bioactive compound release, thus, plant sterols could be used as an alternative [84].

Pan et al. [109] revealed that the incorporation of astaxanthin into the lipid bilayer decreased membrane fluidity, but increased micropolarity in the membrane within a certain range of astaxanthin concentrations. Additionally, it indicated that the encapsulation of astaxanthin in the lipid bilayer could be applied to modulate the structural properties of membranes. Tan et al. [110] found that selected carotenoids exhibited various loading abilities into liposomes ranging from the strongest to the weakest: lutein > β-carotene > lycopene > canthaxanthin. The storage stability and in vitro release into simulated gastriointestinal (GI) media were also investigated. Storage measurements demonstrated that a liposomal membrane can strongly retain β-carotene and lutein and in vitro release experiments showed that lycopene and canthaxanthin underwent fast and considerable release in GI media. It was further revealed [111] that bioaccessibility of carotenoids after encapsulated in liposomes was in the order lutein > β-carotene > lycopene > canthaxanthin and depended strongly on the incorporating ability of carotenoids into a lipid bilayer, loading content, and nature of the system. A third study [112] was conducted to understand how these carotenoids exerted antioxidant activity after encapsulation in the liposome. The encapsulation enhanced the antioxidant activity in different antioxidant models. The strongest antioxidant activity was observed with lutein, followed by β-carotene, lycopene, and canthaxanthin, a similar trend compared to the loading ability and bioaccessibility. Furthermore, lipid peroxidation model revealed that the incorporation of either lutein or β-carotene not only exerts strong lipid peroxidation inhibition capacity during liposome preparation but also protects them against pro-oxidation, while for lycopene and canthaxanthin this effect was weak or a pro-oxidation effect even appeared, concomitantly leading to the considerable depletion of these encapsulated carotenoids. In another research study, methotrexate-induced kidney dysfunction was more efficiently reduced by treatment with lycopene encapsulated nanoliposome comparing to lycopene in standard vehicle [113].

### 3.3. Lipid-Based Nanoparticles: Solid Lipid Nanoparticles (SLNs) and Nanostructured Lipid Carriers (NLCs)

Solid lipid nanoparticles (SLNs) and nanostructured lipid carriers (NLCs) are two subgroups of lipid-based nanoparticles [19,20]. Lipid-based nanoparticles have a lipophilic matrix structure consisting of biocompatible and biodegradable lipids which are completely or mainly solid at room temperature [114]. The classification of SLNs and NLCs is according to their internal structure. SLNs are constituted only by solid lipids, whereas NLCs are formulated by a mixture of mainly solid in combination with liquid lipids (see Figure 8).

Lipid-based nanoparticles have potential applications in drug and food delivery, cosmetics, clinical medicine, pharmaceutical research, gene therapy, etc [115,116]. SLNs were developed in early 1990s to combine the advantages and overcome the limitations of conventional lipid-based systems such as nanoemulsions or nanoliposomes because they have good release control ability and targeted drug delivery with excellent physical stability [116,117]. Although numerous conventional lipid-based systems provided positive results, some reports suggested that these systems are susceptible to degradation during storage and digestion [118].

#### 3.3.1. Solid Lipid Nanoparticles (SLNs)

SLNs are generated by exchanging the liquid core (oil), composed of a conventional oil-in-water (O/W) emulsion, for a solid lipid such as triglycerides, glyceride mixtures, or waxes (see Figure 8). SLNs are composed of approximately 0.1–30 (% *w*/*w*) solid fat dispersed in an aqueous phase and surfactants are used in concentrations of about 0.5 to 5% to enhance stability. The proper selection of lipids and surfactants can affect the particle size, stability during storage, loading capacity and release control ability [116].

SLNs have emerged as promising drug delivery systems in cancer therapy [119] and could be an effective and promising strategy to improve the biopharmaceutical properties of carotenoids for anticancer effects [120]. Table 5 shows current approaches in carotenoid-loaded SLNs reported in references 120–124).

β-Carotene-loaded SLNs for enhanced bioavailability and therapeutic efficacy were prepared, characterized in terms of particle size, morphology, release behavior, and stability, and studied for in vitro cytotoxicity in human breast cancer cell lines and pharmacokinetic studies in Wistar rats. An improved anticancer activity was observed in β-carotene loaded SLNs as compared to the free carotenoid, which in free form was severely affected due to its low bioavailability and oxidative degradation [120]. Lycopene is another carotenoid that shows good pharmacological properties including antioxidant, anti-inflammatory and anticancer. Nazemiyeh et al. [121] have prepared and characterized lycopene-loaded SLNs which were stable in aqueous medium for two months making this system a possible candidate for the future in vivo trials in nutraceutical industries. Bixin was also successfully loaded into SLNs and characterized including in vitro drug release and in vivo evaluation of hepatoprotective activity using Wistar rats. In vivo studies revealed better treatment of paracetamol induced hepatotoxicity by bixin SLNs [122]. Another study, both in vivo in Wistar rats and in vitro in a system consisting of neuronal cell lines treated with H_2_O_2_ to induce cellular injury, investigated the intranasal delivery of astaxanthin SLNs as compared to the intravenous route to improve brain targeting of astaxanthin for neurological disorders. The astaxanthin-loaded SLNs have shown a strong neuroprotective effect against oxidative stress induced in neuronal cell lines [123].

Yi et al. [124] investigated different protein surfactants such as sodium caseinate (SC), whey protein isolate (WPI), or soy protein isolate (SPI) and showed that protein stabilized β-carotene-loaded SLNs can improve stability and uptake of β-carotene. SC was the most efficient surfactant in terms of oxidation stability of loaded β-carotene and provided the highest retention rates of β-carotene in nanoparticles after 30 days of storage at 25 °C, while WPI included in the delivery system provided the most uptake of β-carotene (3.4-fold) versus free β-carotene.

#### 3.3.2. Nanostructured Lipid Carriers (NLCs)

The next generation of lipid nanoparticles, NLCs, has been introduced at the end of the 1990s to overcome some of the problems of SLNs like poor loading capacity and release control. They are modified SLNs which incorporate at least a liquid lipid moiety in the solid lipid lattice. This improves the stability, the low capacity loading caused by the rigid structure, and provides better release control [85,118]. NLCs are produced by dispersion of mixture of solid and liquid lipids, and active ingredients (as inner phase) in water containing emulsifiers (as outer phase) (see Figure 8c). In contrast to SLNs, the presence of liquid lipid in inner phase of NLCs provides possibility of entrapping active ingredients (see Table 6 for recent results of NLCs used as delivery system for carotenoid encapsulation) which are better solubilized in liquid lipid [117,118]. Less ordered structures are produced, which offer the firmer inclusion of the active molecules within the matrix during the shelf life. Higher loading capacity, long shelf storage stability, feasibility of incorporating both hydrophilic and lipophilic drugs, along with possible sustained release and cell targeting make NLCs advanced carriers in comparison to other conventional lipid-based systems [118]. Different research studies in the past decade have shown that NLCs are promising tools for enhancing therapeutic efficacy of drugs, and also for providing controlled release of encapsulated drugs, however, detailed in vivo studies are mandatory for taking them to clinics. The increased research on this delivery system to be used for oral delivery will definitely reach clinical studies in near future [118,125].

Despite their bioactive properties, compounds derived from plants, or phytochemicals (carotenoids among them), are still not commonly used in clinical practice due to several reasons, mainly attributed to their poor bioavailability. In this sense, new formulation strategies are proposed as carriers to improve their bioefficacy, highlighting the use of lipid-based delivery systems [126]. However, research on developing and testing such systems is scarce. Astaxanthin is known to possess potential antitumoral activity, yet it is currently studied in vitro and in animals against a variety of animal- and human-cancer cell lines [127], far yet from reaching clinical studies. There are currently no studies on incorporating astaxanthin in SLNs. Lycopene is also natural chemo-preventative agent and possesses anti-cancer cell proliferation activity for prostate, breast and skin cancers. A SLN for encapsulation of lycopene was developed and shown to improve oral delivery. Upon ex vivo assessment it was determined that lycopene-loaded NLCs had better permeation and caused more cytotoxicity against the human breast tumor cells [128].

Applications in food are possibly more promising. Tamjidi et al. developed a stable NLC for incorporation of astaxanthin [129] that was used in various model beverages [130]. Results showed that NLCs containing hydrophobic nutraceuticals have potential to be used for beverages or foods applications. A similar delivery system by Rodriguez-Ruiz [131] in which astaxanthin was incorporated in NLCs preserved and enhanced the antioxidant activity of the carotenoid. Other carotenoids such as β-carotene [132] or lycopene [133] were incorporated in NLCs; these delivery systems increased the stability of the carotenoids during a couple and a few months period, respectively.

## 4. Biopolymeric Nanoparticles

Nanoencapsulation of carotenoids with different nanocarriers could improve their stability and bioavailability and increase their application. The structures and compositions of delivery systems play significant roles in the bioaccessibility of carotenoids during digestion. Besides lipid-based nanocarriers, biopolymeric nanoparticles are also commonly used to encapsulate carotenoids. They can be carbohydrate or protein-based and they possess advantages like biodegradability, bioavailability and relatively low cost.

Biopolymeric nanoparticles range in size from about 1–1000 nanometers in diameter, about one thousand times smaller than the average cell in a human body [134]. They are mainly developed for drug delivery systems as an alternative to liposomes because they offer advantages of drug targeting as well as the enhancement of the cellular uptake [135]. Thus, biopolymeric nanoparticles hold applications as delivery systems for administration of drugs and vaccines because the bioactive molecules can be delivered at specific targeted sites.

Biopolymeric nanoparticles are based on synthetic polymers, carbohydrates or proteins, or their nanocomplexes [135], and they can encapsulate hydrophobic compounds such as carotenoids. Among the synthetic polymers, approved by the FDA, the most commonly used are polylactic acid (PLA), poly-L-lysine (PLL), polyglutamic acid (PGluA), polyglycolic acid (PGA), polyethylene glycol (PEG), polycaprolactone (PCL), polyaspartate (PAA), poly(D,L-lactide-*co*-glycolic) acid (PLGA), cyclodextrins (CD), and *N*-(2-hydroxypropyl)-ethacrylamide copolymer (HPMA) [136]. Among the natural polymers, proteins [134] like gelatin and albumin, and polysaccharides like alginate and chitosan are frequently used because they are abundant in nature, biodegradable and relatively inexpensive.

Chitosan is the most common ingredient in formulating biopolymeric nanoparticles for carotenoid encapsulation (see Table 7 for a summary of the results in [137,138,139,140,141]). It is an excellent biopolymer for preparation of nanoparticles due to its excellent biocompatibility and biodegradability. Stability studies showed that nanoencapsulation provided enhanced crocin stability with chitosan-sodium alginate nanoparticles compared to the standard crocin under unfavorable environmental conditions [137]. Water-soluble chitosan/poly-γ-glutamic acid (γ-PGA) nanoencapsulation was used to enhance the solubility of lutein [138]. Astaxanthin was encapsulated in poly(lactic-co-glycolic acid) (PLGA) nanoparticles coated with chitosan oligosaccharides (COS) [139].

Zein, a naturally occurring protein derived from corn, used to produce nanoparticles for incorporating carotenoids, is also often mentioned in the literature (see Table 8 for a summary of the reported results). Due to the protein’s hydrophobicity, zein nanoparticles are promising oral drug delivery vehicles able to protect encapsulated bioactive compounds from harsh acidic environments such as gastric acid [134]. Lutein encapsulated in zein nanoparticles improved digestive stability of the carotenoid in gastric conditions and provided sufficient nutritive lutein content [142]. Combining the natural zein polymer with other substances can improve their properties [134]. For example, zein was used in combination with sodium caseinate [143] and with poly(lactic-co-glycolic acid) (PLGA) [144] to encapsulate carotenoids. While sodium caseinate is a food-grade protein, poly PLGA is a synthetic polymer, and makes one of the most effective biodegradable polymeric nanoparticles approved by the FDA to use in drug delivery systems due to controlled and sustained-release properties, low toxicity, and biocompatibility with tissue and cells [145].

Fucoxanthin encapsulated in zein caseinate nanoparticles showed thermal and storage stability, and improved bioaccessibility, stronger antioxidant and anticancer activity than free fucoxanthin [143]. Lutein in zein poly(lactic-co-glycolic acid) nanoparticles delivered more of this naturally occurring carotenoid in the eye via topical application and significantly reduced cataract in rats compared to free lutein used in oral and topical formulations [144]. In another study, zein nanoparticles stabilized with surfactants protected lutein against thermal and UV degradation and slowed down its release [146]. β-Carotene-loaded zein nanoparticles produced an improvement in oral bioavailability and cytotoxicity, and an enhanced biopharmaceutical performance of β-carotene indicating that this carotenoid has a protective role on methotrexate-associated hepatic toxicity in Wistar rats [147].

## 5. Conclusions

Carotenoids are hydrophobic, chemically unstable molecules, highly reactive being prone to oxidation. The presence of conjugated double bonds in carotenoid structure contributes for their pigmentation, absorption of UV-Vis radiation, and antioxidant activity but also is the main reason of their chemical instability. 

The efficient way to increase the bioavailability of carotenoids is the increase of their solubility and stability by encapsulation into water-soluble carriers like inclusion complexes or into biodegradable delivery systems such as lipid-, protein- or carbohydrate-based nanocarriers. Encapsulation of the active compound can enhance its efficiency, specificity and targeting ability. At this moment there are already a large number of examples indicating that encapsulation of lipophilic carotenoids within nanocarriers as delivery systems is the approach that increases their solubility, stability and bioavailability features important for practical applications. We have found that encapsulation affects many fundamental physical and chemical properties of carotenoids, including optical properties, self-association ability, oxidation potentials, stability of paramagnetic forms, and many others described in the present review.

As a conclusion, we can summarize that there is no universal multifunctional carrier able to improve all carotenoid features simultaneously. Most of delivery systems improve carotenoids solubility, especially by incorporation into water-soluble carriers like CD, AG or GA. Some of the carriers improve carotenoids stability during the storage and food processing, as well as their bioavailability in vivo experiments, both oral and topical delivery routes. Only a few examples exist on increasing photostability of carotenoids in inclusion complexes. Importantly, some physicochemical studies provide evidence on the effect of encapsulation on fundamental properties of carotenoids, redox potentials, quantum yields and life time of excited states, optical properties (absorption and fluorescence spectra), as well as properties of paramagnetic forms of carotenoids.

## Figures and Tables

**Table 1:** Relative scavenging rate constants of OOH radicals by carotenoids and their GA complexes (k/k_ST_) in DMSO. E_1/2_ is the redox potential of carotenoids (in Volts vs. SCE) referenced to ferrocene. [17,59].

[GA], mM	β-Carotene	Canthaxanthin	7-*apo*-7,7-Dicyano-β-carotene
	(E_1/2_ = 0.634 V)	(E_1/2_ = 0.765 V)	(E_1/2_ = 0.825 V)
0	0.5	2	7
0.5	0.5	59	133

**Table 2:** Inclusion complexes of cyclodextrin (CD), glycyrrhizic acid (GA) and arabinogalactan (AG) as delivery systems for carotenoid encapsulation.

Delivery System	Carotenoid	Encapsulation Method	Results	Reference
β-CD	lutein, zeaxanthin, α-cryptoxanthin, α-carotene and β-carotene	kneading method	better color protection for the beverage	[30] Lobo 2018
β-CD	lycopene, lutein, capsanthin and capsorubin	mixture of methylene chloride solution of carotenoid with ethanol solution of the carrier.	more stable against oxidating agents as AAPH and H_2_O_2_	[31] Fernández-García 2017
β-CD	bixin	freeze-drying technique	more palatable and hepatoprotective effect	[32] Pinzón-García 2018
methyl-β-CD	astaxanthin	spray drying method	improved solubility	[33] Nalawade 2015
GA, AG	astaxanthin, lutein, zeaxanthin	mixture of ethanol solution of carotenoid with water solution of the carrier.	solubility enhancement, prevention of H-aggregates formation in ethanol/water mixture, 7 folds increase of photostability in solution.	[16] Polyakov 2006 [59] Polyakov 2013
GA, Na_2_GA or AG	lutein, zeaxanthin	solid state mechanochemical method	2000 fold solubility enhancement, more than 10 fold increase of carotenoids stability in solution towards oxidation by ozone and Fe ions	[60] Apanacenko 2015
GA and AG	lutein, zeaxanthin, and β-carotene	solid state mechanochemical method	solubility and bioavailability enhancement, increase of zeaxanthin level in the serum, liver and RPE/choroid of mice.	[61] Li 2017

**Table 3:** Nanoemulsions as delivery system for carotenoid encapsulation.

Delivery System	Carotenoid	Encapsulation Method	Results	Reference
Tween 20 stabilized O/W emulsion	β-carotene	high-pressure homogenization	higher bioaccessibility in low fat oil	[90] Xia 2017
whey protein isolate (WPI) stabilized O/W nanoemulsion	β-carotene	high-pressure homogenization	higher bioaccessibility of β-carotene with palm oil	[93] Zhou 2018
caseinate stabilized nanoemulsions	astaxanthin	pressure homogenization	emulsion stable at all incubation temperatures (5–70 °C) and chemical stability of astaxanthin against changes in pH, ionic strength and light exposure	[98] Liu 2016
sodium caseinate stabilized O/Wnanoemulsions	β-carotene	homogenization at low and high pressures	very stable to coalescence or flocculation over 30 days	[99] Yi 2014
Whey protein stabilized O/W nanoemulsion	lutein	ultrasonication	encapsulated lutein content was reduced by only 4% after four weeks storage at 4 °C	[100] Zhao 2019
modified lecithin (ML) versus sodium caseinate (SC) stabilized O/W nanoemulsions	astaxanthin	high-pressure homogenization	good physical and chemical stability (>70%) after 30 days of storage for SC	[101] Khalid 2017
Ginseng saponins stabilized O/W nanoemulsions	astaxanthin	high-pressure homogenization	excellent stability at 5 °C	[105] Shu 2018
gypenosides stabilized O/W nanoemulsions	astaxanthin	high-pressure homogenization	stability during 30 days of storage at both 5 and 25 °C	[106] Chen 2018
O/W nanoemulsion stabilized with bovine serum albumin (BSA) as the inner emulsifier and Arabic gum as outer emulsifier	β-carotene	high-pressure homogenization	better chemical stability under different environmental stresses when compared with monolayer emulsions studied	[108] Sheng 2018

**Table 4:** Nanoliposomes as delivery system for carotenoid encapsulation.

Delivery System	Carotenoid	Encapsulation Method	Results	Reference
soybean phosphatidyl choline nanoliposomes	astaxanthin	film dispersion-ultrasonic technique	enhanced thermal stability and water dispersibility of astaxanthin.	[109] Pan 2018
nanoliposomes	lutein, β-carotene, lycopene, and canthaxanthin.	thin-film evaporation method	enhanced loading ability, bioaccesibility and antioxidant activity in this order: lutein> β-carotene> lycopene> canthaxanthin	[110,111,112] Tan 2014
phospholipid nanoliposomes	lycopene	centrifugation	enhanced antioxidant activity, preventing reactive oxygen species-induced kidney tissue damage	[113] Stojiljkovic 2018

**Table 5:** Solid lipid nanoparticles (SLNs) as delivery system for carotenoid encapsulation.

Delivery System	Carotenoid	Encapsulation Method	Results	Reference
SLNs	β-carotene	hot homogenization	improved bioavailability and anticancer activity	[120] Jain 2019
SLNs	lycopene	modified hot homogenization method	stable after 2 months in aqueous medium (4 °C)	[121] Nazemiyeh 2016
soya and egg lecithin stabilized SLNs	bixin	hot homogenisation followed by ultrasonication technique	entrapment efficiency, loading efficiency and enhanced hepatoprotection	[122] Rao 2014
Poloxamer 188 and lecithin stabilized SLNs	astaxanhin	solvent displacement method	neuroprotection from oxidative stress	[123] Bhatt 2016
sodium caseinate (SC), whey protein isolate (WPI), or soy protein isolate (SPI) stabilized SLNs	β-carotene	homogenization-evaporation method	improved stability and uptake of betacarotene	[124] Yi 2014

**Table 6:** Nanostructured lipid carriers (NLCs) as delivery system for carotenoid encapsulation.

Delivery System	Carotenoid	Encapsulation Method	Results	Reference
Tween 80 and Poloxamer 188 stabilized NLCs	lycopene	ultrasonication	enhanced oral bioavailability of lycopene, increased cytotoxicity against the human breast tumour cells	[128] Singh 2017
Tween 80 and lecithin stabilized NLCs	astaxanthin	melt-emulsification and ultrasonication technique	pH, ionic strength, heat and simulated gastric juice had no drastic effects on the chemical stability	[129] Tamjidi 2017
Tween 80 stabilized NLCs	astaxanthin	melt-emulsification and ultrasonication technique	improved the physical stability in acidic beverage (solutions with 0 or 12% sucrose; pH 3–7) during 30–60 days storage at 6 or 20 °C.	[130] Tamjidi 2017
Tween 80 and Poloxamer 407 stabilized NLCs	astaxanthin	hot homogenization (HH) method	loading capacity (90%) and enhanced antioxidant activity	[131] Rodriguez-Ruiz
Poloxamer 407 (3%) stabilized NLCs	β-carotene	hot-high shear homogenizer (Hot-HSH) method	stable after 60 days of storage at 25 °C	[132] Zirak 2015
Eumulgin SG stabilized NLCs	lycopene	high pressure homogenization	high stability at different temp. (4, 30, 40 °C) for 120 days; no degradation at low temperatures.	[133] Okonogi 2015

**Table 7:** Polymeric nanoparticles as delivery system for carotenoid encapsulation: chitosan nanoparticles.

Delivery System	Carotenoid	Encapsulation Method	Results	Reference
chitosan/sodium alginate	crocin	modified ionic gelation method	better stability of nanoparticles during manipulation and storage.	[137] Rahaiee 2015
chitosan/poly-glutamic acid	lutein	ionic gelation	12 fold solubility enhancement	[138] Hong 2016
chitosan oligosaccharides and poly(lactic-co-glycolic acid)	astaxanthin	spontaneous self-assembly	improved stability and prolonged release in simulated gastrointestinal juices	[139] Liu 2019
chitosan- nanoparticle	lutein		topical ocular delivery	[140] Chaiyasan 2015
DNA/chitosan nanoparticles	astaxanthin		improved cellular uptake and antioxidation capability	[141] Wang 2017

**Table 8:** Polymeric nanoparticles as delivery system for carotenoid encapsulation: zein nanoparticles.

Delivery System	Carotenoid	Encapsulation Method	Results	Reference
zein nanoparticles	lutein	solvent-induced nanoprecipitation	improved lutein digestive stability but reduced micellarization	[142] Cheng 2018
zein-caseinate nanoparticles	fucoxanthin	antisolvent precipitation	increased stability, bioaccessibility, antioxidant activity and antiproliferative activity	[143] Li 2018
zein/ poly(lactic-co glycolic acid) nanoparticles	lutein	modified emulsion/evaporation method	enhanced stability and increased bioavailability of lutein in the rat eye when delivered topically	[144] Bodoki 2018
zein nanoparticles with lecithin and pluronic F127 surfactants	lutein	liquid-liquid dispersion method	increased stability and controlled release	[146] Chuacharoen 2016
zein with Tween 80 and pluronic F-68 surfactants	β-carotene	modified phase separation technique	Improved cellular uptake, cytotoxicity and oral biopharmaceutical performance	[147] Jain 2018
